# Mutations of the **β**- and **γ**-catenin genes are uncommon in human lung, breast, kidney, cervical and ovarian carcinomas

**DOI:** 10.1054/bjoc.2001.1863

**Published:** 2001-07

**Authors:** M Ueda, R M Gemmill, J West, R Winn, M Sugita, N Tanaka, M Ueki, H A Drabkin

**Affiliations:** Department of Obstetrics and Gynecology, Osaka Medical College, 2-7 Daigakumachi, Takatsuki, Osaka 569–8686, Japan; University of Colorado Health Sciences Center, Division of Medical Oncology and; Department of Pathology, 4200 East 9th Avenue, Denver, CO 80262, USA; Department of Obstetrics and Gynecology, Chiba University School of Medicine, 1-8-1, Inohana, Chuo-ku, Chiba 260-8670, Japan

**Keywords:** β-catenin, γ-catenin, gene mutation, human carcinoma

## Abstract

β-catenin forms complexes with Tcf and Lef-1 and functions as a transcriptional activator in the Wnt signalling pathway. Although recent investigations have been focused on the role of the adenomatous polyposis coli (APC)/ β-catenin/Tcf pathway in human tumorigenesis, there have been very few reports on mutations of the β-catenin gene in a variety of tumour types. Using PCR and single-strand conformational polymorphism analysis, we examined 93 lung, 9 breast, 6 kidney, 19 cervical and 7 ovarian carcinoma cell lines for mutations in exon 3 of the β-catenin gene. In addition, we tested these same samples for mutations in the NH_2_-terminal regulatory region of the γ-catenin gene. Mutational analysis for the entire coding region of β-catenin cDNA was also undertaken in 20 lung, 9 breast, 5 kidney and 6 cervical carcinoma cell lines. Deletion of most β-catenin coding exons was confirmed in line NCI-H28 (lung mesothelioma) and a silent mutation at codon 214 in exon 5 was found in HeLa (cervical adenocarcinoma). A missense mutation at codon 19 and a silent mutation at codon 28 in the NH_2_-terminal regulatory region of the γ-catenin gene were found in H1726 (squamous cell lung carcinoma) and H1048 (small cell lung carcinoma), respectively. Neither deletions nor mutations of these genes were detected in the other cell lines examined. These results suggest that β- and γ-catenins are infrequent mutational targets during development of human lung, breast, kidney, cervical and ovarian carcinomas. © 2001 Cancer Research Campaign http://www.bjcancer.com


*British Journal of Cancer* (2001) **85**(1), 64–68
© 2001 Cancer Research Campaign
doi: 10.1054/ bjoc.2001.1863, available online at http://www.idealibrary.com on IDEAL®

### Mutations of the β- and γ-catenin genes are uncommon in human lung, breast, kidney, cervical and ovarian carcinomas

**M Ueda[1], RM Gemmill[2], J West[2], R Winn[2], M Sugita[3], N Tanaka[4], M Ueki[1] and HA Drabkin[2]**

[1]Department of Obstetrics and Gynecology, Osaka Medical College, 2-7 Daigakumachi, Takatsuki, Osaka 569–8686, Japan; [2]University of Colorado Health Sciences Center, Division of Medical Oncology and [3]Department of Pathology, 4200 East 9th Avenue, Denver, CO 80262, USA; [4]Department of Obstetrics and Gynecology, Chiba University School of Medicine, 1-8-1, Inohana, Chuo-ku, Chiba 260-8670, Japan

**Summary** β-catenin forms complexes with Tcf and Lef-1 and functions as a transcriptional activator in the Wnt signalling pathway. Although recent investigations have been focused on the role of the adenomatous polyposis coli (APC)/ β-catenin/Tcf pathway in human tumorigenesis, there have been very few reports on mutations of the β-catenin gene in a variety of tumour types. Using PCR and single-strand conformational polymorphism analysis, we examined 93 lung, 9 breast, 6 kidney, 19 cervical and 7 ovarian carcinoma cell lines for mutations in exon 3 of the β-catenin gene. In addition, we tested these same samples for mutations in the $NH_2$-terminal regulatory region of the γ-catenin gene. Mutational analysis for the entire coding region of β-catenin cDNA was also undertaken in 20 lung, 9 breast, 5 kidney and 6 cervical carcinoma cell lines. Deletion of most β-catenin coding exons was confirmed in line NCI-H28 (lung mesothelioma) and a silent mutation at codon 214 in exon 5 was found in HeLa (cervical adenocarcinoma). A missense mutation at codon 19 and a silent mutation at codon 28 in the $NH_2$-terminal regulatory region of the γ-catenin gene were found in H1726 (squamous cell lung carcinoma) and H1048 (small cell lung carcinoma), respectively. Neither deletions nor mutations of these genes were detected in the other cell lines examined. These results suggest that β- and γ-catenins are infrequent mutational targets during development of human lung, breast, kidney, cervical and ovarian carcinomas. © 2001 Cancer Research Campaign http://www.bjcancer.com

**Keywords**: β-catenin; γ-catenin; gene mutation; human carcinoma

β-catenin, the vertebrate homologue of *Drosophila* Armadillo, is a multifunctional protein involved in 2 apparently independent processes, acting as a cell–cell adhesion regulator when coupled with cadherins (Barth et al, 1997) and as a transcriptional regulator in the wingless/Wnt signal transduction pathway (Behrens et al, 1996). β-catenin forms complexes with Tcf and Lef-1 and functions to transactivate downstream target genes of the Wnt pathway (Behrens et al, 1996). Activation of the pathway by stabilization of β-catenin has been shown to be important in the development of colorectal carcinoma, which is mainly caused by inactivating mutations of the adenomatous polyposis coli (APC) tumour suppressor gene or by activating mutations in exon 3 of the β-catenin gene that includes the glycogen synthase kinase-3β (GSK-3β) phosphorylation site (Morin et al, 1997; Sparks et al, 1998). Mutations of the β-catenin gene have recently been implicated in the initiation of melanomas and some colorectal carcinomas (Morin et al, 1997; Rubinfeld et al, 1997; Sparks et al, 1998). Although recent investigation have been focused on the role of the APC/β-catenin/Tcf pathway in human tumorigenesis, there have been very few reports on mutations of the β-catenin gene in a variety of tumour types except for melanomas (Rubinfeld et al, 1997), colorectal (Kitaeva et al, 1997; Morin et al, 1997; Sparks et al, 1998) and endometrioid carcinomas (Fukuchi et al, 1998; Palacios and Gamallo, 1998). γ-catenin has also been linked to the APC/β-catenin/Tcf pathway by biochemical and genetic studies in diverse organisms (Peifer, 1996). γ-catenin shares strong amino acid similarity with β-catenin and the $NH_2$-terminal regulatory motif is conserved between the 2 proteins. In addition, both proteins bind APC, α-catenin, E-cadherin and Tcf (Rubinfeld et al, 1995; Sacco et al, 1995). Moreover, γ-catenin exhibits signaling activity similar to that of β-catenin in *Xenopus* (Karnovsky and Klymkowsky, 1995), and functions as an oncogene (Kolligs et al, 2000). Therefore, activating mutations in the Wnt signalling pathway could reasonably be expected to occur in the γ-catenin gene. In this report, we analysed the β- and γ-catenin genes for mutations in a large series of human lung, breast, kidney, cervical and ovarian carcinoma cell lines. Our findings suggest mutations in these genes are quite rare among the tumour types examined.

*Received 21 November 2000*
*Revised 5 March 2001*
*Accepted 20 March 2001*

*Correspondence to:* M Ueda

### MATERIALS AND METHODS

### DNA preparation

Analyses were conducted on 93 lung, 9 breast, 6 kidney, 19 cervical and 7 ovarian carcinoma cell lines (Table 1). All lung, breast, kidney and 7 (ME180, SiHa, CC19, Caski, C-33A, C-4i, HeLa) cervical carcinoma cell lines were obtained from the American Type Culture Collection. The other 12 cervical and all ovarian carcinoma cell lines were kindly provided from the Japanese institutes where each cell line was established. Detailed information for cell lines examined is available from the authors upon request. These cell lines were grown either in RPMI 1640 or

**Table 1** Human lung, breast, kidney, cervical and ovarian carcinoma cell lines examined

| Lung carcinoma | | | Breast carcinoma | Cervical carcinoma |
|---|---|---|---|---|
| Small cell | H2018 | H1155 | CRL1504[a] | Squamous cell carcinoma |
| | H2107 | H1264 | HTB121[a] | |
| H69 | H2141 | H1155 | HTB122[a] | SKG-I |
| H82[a] | H2171 | H1299 | HTB124[a] | SKG-II |
| H187[a] | H2195 | H1334[a] | HTB132[a] | SKG-IIIa |
| H312 | H2196 | H1385 | MCF-7[a] | QG-H |
| H345 | H2227 | H1395 | MDA231[a] | QG-U |
| H378 | SHP77 | H1404 | T470[a] | YUMOTO |
| H446 | COLO668 | H1437 | ZR75.1[a] | HOKUG |
| H524 | COLO699 | H1438 | | ME180[a] |
| H526 | NA17 | H1573 | **Kidney carcinoma** | SiHa |
| H660 | NE18 | H1581[a] | | CC19[a] |
| H711 | NU6-1 | H1623 | A498 | Caski[a] |
| H719 | umc19 | H1648[a] | PV10[a] | C-33A[a] |
| H735 | UCLC11 | H1650 | KV6[a] | C-4i[a] |
| H748 | 6LC20[a] | H1693 | ACHN[a] | |
| H792 | | H1726 | KRC-Y[a] | Adenocarcinoma |
| H865 | Non small cell | H1781 | Caki2[a] | |
| H889 | | H1792 | | NUZ |
| H1045 | H28[a] | H1819 | | CAC-1 |
| H1048[a] | H125 | H1869 | | OMC-4 |
| H1062 | H226[a] | H2009 | | TCO-1 |
| H1092 | H290[a] | H2052 | | TCO-2 |
| H1105 | H292[a] | H2058 | | HeLa[a] |
| H1238 | H320 | H2077 | | |
| H1284 | H324 | H2087 | | **Ovarian carcinoma** |
| H1304 | H460[a] | H2122[a] | | |
| H1339 | H513[a] | H2258 | | HOC-1 |
| H1341[a] | H522 | NCIA549[a] | | HOC-21 |
| H1417 | H661[a] | | | HAC-2 |
| H1450 | H720[a] | | | SHIN-3 |
| H1514 | H727[a] | | | HVOCA-II |
| H1522 | H810 | | | HMOA |
| H1607[a] | H838 | | | HUOA |
| H1618 | | | | |
| H1876 | | | | |

[a]Total RNAs were extracted and cDNAs were prepared for RT-PCR-SSCP analysis.

McCoy's medium (Sigma) supplemented with 10% or 15% fetal calf serum at 37˚C in 5% $CO_2$ atmosphere and were actively growing prior to harvesting. The cells were disrupted with lysis buffer (20 mM NaCl, 10 mM Tris-HCl (pH 8.0), 10 mM EDTA (pH 8.0), 0.5% SDS, 50 µg $ml^{-1}$ proteinase K), and then genomic DNA was extracted with phenol-chloroform and precipitated with ethanol using standard techniques.

### RNA isolation and cDNA preparation

RNA was extracted from 20 lung, 9 breast, 5 kidney and 6 cervical carcinoma cell lines (Table 1) by a combination of initial phenol/chloroform extraction according to the RNA STAT-60 protocol (Tel-Test, Inc) and then SV-total RNA isolation kit extraction (Promega, Inc) according to the supplier's protocol. Contaminating residual genomic DNA was removed by digestion with RNase free DNase. cDNAs were prepared using at least 2 µg of total RNA and SUPERSCRIPT II reverse transcriptase (Life Technologies, Inc) with random hexamers as primers.

### PCR for β- and γ-catenins

For β-catenin, a genomic PCR fragment including codons 6–80 in exon 3 encompassing the GSK-3 β phosphorylation site was amplified (primers: forward, 5′-attgatggagttggacatggc-3′; reverse, 5′-ccagctacttgttcttgagtgaagg-3′; Kitaeva et al, 1997). For γ-catenin, a genomic fragment encoding amino acids 1–57 of γ-catenin and encompassing the $NH_2$-terminal regulatory region was amplified (primers: forward, 5′-ctcagtagccacgatggaggtg-3′; reverse, 5′-ttcttgagcgtgtactggcg-3′; Sparks et al, 1998). One µl of the DNA template was amplified by PCR in a 50 µl reaction containing 10 mM Tris-HCl (pH 8.3), 50 mM KCl, 2 mM $MgCl_2$, 0.01% (w/v) gelatin, 200 µM dNTP, 0.5 µM each primer and 1.25 units Taq polymerase (Perkin Elmer Cetus). PCR was carried out on a Perkin-Elmer GeneAmp PCR System 9600 with an initial denaturation step at 96˚C for 3 min then 35 cycles at 94˚C for 1 min, 55˚C for 1 min, 72˚C for 2 min, and a final extention cycle at 72˚C for 10 min. Reaction products were visualized on a 1.5% agarose gel with ethidium bromide.

### Reverse transcription (RT)-PCR for β-catenin

After RT of mRNA, 1 µl of the cDNA template was amplified by PCR as described above using 6 primer pairs which cover the entire coding region of the β-catenin cDNA. Detailed PCR conditions and primer sequences used for amplification are available from the authors upon request. Reaction products were visualized as described above.

*© 2001 Cancer Research Campaign* 

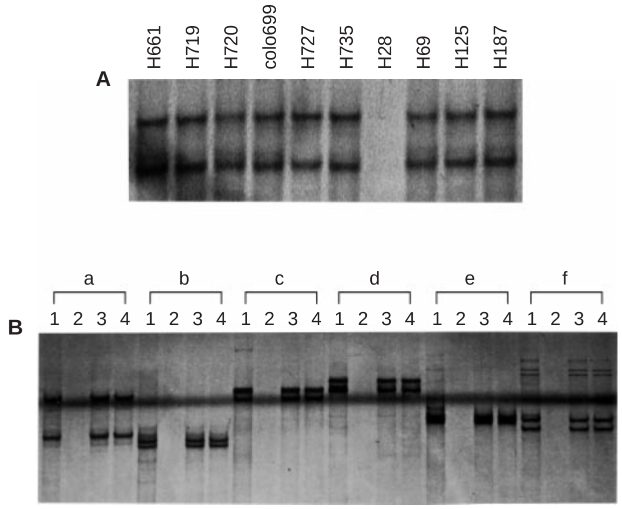

**Figure 1** Mutational analysis of the β-catenin gene in lung carcinoma cells. **A**, SSCP analysis of the PCR amplification of β-catenin exon 3 in 10 lung carcinoma cell lines. Deletion of exon 3 of the β-catenin gene was found in NCI-H28 (lung mesothelioma). **B**, SSCP analysis of the RT-PCR amplification products from β-catenin exons 2–16 in 3 lung mesothelioma cell lines and normal bronchial epithelial cells. Lanes 1–4 correspond to samples from NCI-H513, NCI-H28, NCI-H290 (lung mesothelioma) and PDL6 (normal bronchial epithelial cells), respectively. RT-PCR amplification was conducted using 6 primer pairs which cover the entire coding region of the β-catenin cDNA (a, exons 2–4; b, exons 4–6; c, exons 6–9; d, exons 8–10; e, exons 10–13; f, exons 13–16, respectively). All products are missing from NCI-H28, indicating absence of β-catenin message

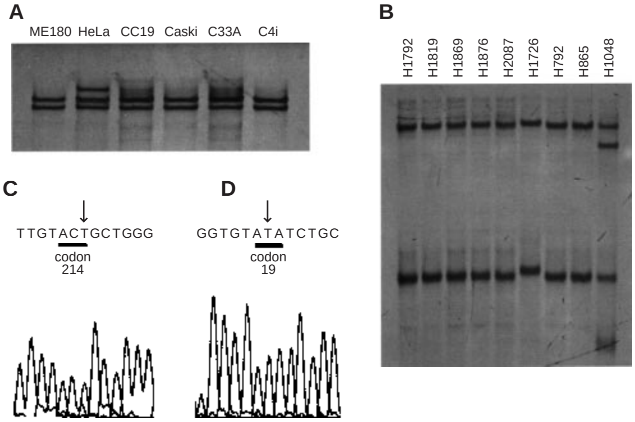

**Figure 2** Mutational analysis of the β-catenin gene in cervical carcinoma cells and the γ-catenin gene in lung carcinoma cells. **A**, SSCP analysis of the RT-PCR amplification products from β-catenin exons 4–6 in 6 cervical carcinoma cell lines. An aberrant band is seen in HeLa (cervical adenocarcinoma) which was excised, reamplified and sequenced. **B**, SSCP analysis of the PCR amplification of γ-catenin $NH_2$-terminal regulatory region in 9 lung carcinoma cell lines. Aberrant bands are seen in NCI-H1726 (squamous cell lung carcinoma) and NCI-H1048 (small cell lung carcinoma). **C**, Electropherogram of β-catenin forward sequence in HeLa, indicating the silent mutation at codon 214 in exon 5 (ACC to ACT, both coding for Thr). **D**, Electropherogram of γ-catenin reverse sequence in NCI-H1726, indicating the missense mutation at codon 19 (ACA to ATA, Thr to Ile)

### Single-strand conformational polymorphism (SSCP) analysis

The amplified fragments of the β- and γ-catenin genes were analysed for mutations by SSCP according to the method described by Ilyas et al (1997) with some modifications. 5 μl of PCR or RT-PCR products were mixed with 5 μl of loading buffer (95% formamide, 10 mM NaOH, 20 mM EDTA, 0.02% bromophenol blue, 0.02% xylene cyanol), heat denatured at 95˚C for 5 min, and immediately placed on ice. 8 μl of the mixture was run overnight at constant 8 W power on a 0.5 × Mutation Detection Enhancement (MDE) vertical gel (FMC BioProducts) containing 10% glycerol. The gel was stained by soaking for 15 min in a 0.1% solution of silver nitrate. After two quick washes in distilled water, the gel was incubated in a solution of 0.15% NaOH, 0.01% $NaBH_4$ and 0.15% formaldehyde to visualize the bands.

### Sequence analysis

Polymorphisms in the β- and γ-catenin genes, detected as aberrant SSCP patterns, were sequenced. PCR or RT-PCR amplified DNA fragments were purified using the QIAquick gel extraction kit (QIAGEN) and directly sequenced using an Applied Biosystem model 373 DNA sequencer (Perkin Elmer Cetus) with the primer used for PCR or RT-PCR and a Dye Terminator Cycle Sequencing FS Ready Reaction Kit (Applied Biosystems). Each mutation was verified in both the sense and antisense directions.

### RESULTS

Exon 3 was selected first for mutational analysis because it encodes the $NH_2$-terminal regulatory domain of β-catenin previously found to contain activating mutations (Morin et al, 1997; Rubinfeld et al, 1997; Fukuchi et al, 1998; Palacios and Gamallo, 1998; Sparks et al, 1998). PCR and SSCP analysis of this region in 93 lung, 9 breast, 6 kidney, 19 cervical and 7 ovarian carcinoma cell lines identified no point mutations but confirmed the existence of a homozygous deletion in NCI-H28 (lung mesothelioma) as shown in Figure 1A (Calvo et al, 2000). Neither deletions nor mutations of exon 3 of the β-catenin gene were detected in the other cell lines examined. To further investigate whether or not genetic alterations of β-catenin exist in the complete coding sequence, RT-PCR-SSCP analysis was conducted on 20 lung, 9 breast, 5 kidney and 6 cervical carcinoma cell lines using 6 primer pairs which cover the entire coding region of the β-catenin cDNA. As can be seen in Figure 1B, none of these primers amplified β-catenin sequences from NCI-H28 cDNA, indicating that message is either not made or is rapidly degraded. RT-PCR-SSCP analysis on exons 4–6 also detected a variant band in HeLa (cervical adenocarcinoma) cells (Figure 2A). However, sequencing of the gel purified RT-PCR product revealed a silent mutation (ACC to ACT, Thr to Thr) at codon 214 in exon 5 (Figure 2C). These analyses of over 100 tumours indicate that β-catenin mutations occur only rarely in human lung, breast, kidney, cervical and ovarian carcinomas.

If activation of the APC/β-catenin/Tcf signalling pathway is critical for initiation of these tumour types, mutations in other components of the pathway might be involved in the genesis of the tumours. One candidate for such a component is γ-catenin, which shares strong amino acid similarity with β-catenin and the $NH_2$-terminal regulatory motif is conserved between the 2 proteins. To address this possibility, we performed PCR-SSCP analysis on the $NH_2$-terminal regulatory region (codons 1–57) of γ-catenin. Among 134 cell lines examined, we found only two γ-catenin mutations (Figure 2B); a missense mutation at codon 19 (ACA to ATA, Thr to Ile) in NCI-H1726 (squamous cell lung carcinoma)

 © 2001 Cancer Research Campaign

(Figure 2D) and a silent mutation at codon 28 (TCG to TCA, Ser to Ser) in NCI-H1048 (small cell lung carcinoma). Neither deletions nor mutations of this region were detected in the other cell lines examined.

### DISCUSSION

A current hypothesis suggests that inactivation of APC or activating mutations of β-catenin may have similar functional and tumorigenic consequences in the human colon (Morin et al, 1997; Sparks et al, 1998). Consequently, β-catenin can behave as an oncogene, providing a potential alternative mechanism of initiation in the pathway of colorectal carcinogenesis (Peifer, 1997). While mutations of the APC gene initiate approximately 85% of human colorectal carcinomas, the initiating event remains unknown in the balance of cases (Kinzler and Vogelstein, 1996). Recently, mutations in exon 3 of the β-catenin gene were found in 13 of 27 (48%) colorectal tumours lacking APC mutations, suggesting that β-catenin might be mutated in a significant minority of colorectal tumours (Sparks et al, 1998). Activating mutations in β-catenin were also found in melanoma cell lines, implying that β-catenin may act as an oncogene in a variety of other carcinomas, including those without a clear association with APC mutations (Rubinfeld et al, 1997). However, Kitaeva et al (1997) demonstrated that no mutations were detected in the 8 melanoma cell lines. Moreover, Kitaeva et al (1997) and Candidus et al (1996) reported that β-catenin mutations are uncommon in human colorectal, gastric and breast carcinomas.

In the present study, deletion of exon 3 of the β-catenin gene was confirmed in NCI-H28 lung mesothelioma cells. However, our results also indicate that the β-catenin deletion is sufficiently large to eliminate detectable message. Deletion of the $NH_2$-terminal part of β-catenin was described for 2 cell lines originating from signet ring cell carcinomas of the stomach (Oyama et al, 1994; Kawanishi et al, 1995), however, neither deletions nor mutations of exon 3 of the β-catenin gene were detected in the other 133 cell lines examined. β-catenin mutations within armadillo repeats 2 and 3 and outside the $NH_2$-terminal regulatory domain were also described in two colorectal carcinoma cell lines with mutant APC (Ilyas et al, 1997). Here, we observed loss of expression for β-catenin in NCI-H28 lung mesothelioma cells and a silent mutation at codon 214 in exon 5 in HeLa cervical adenocarcinoma cells. However, no genetic alteration outside exon 3 was found in the other 38 cell lines examined. While the effects of deletion for β-catenin are unknown, but its loss likely precludes activation of the canonical Wnt signalling pathway. Further studies are needed to clarify the molecular and biological consequences of this deletion for NCI-H28 cells.

The above analyses and previous reports indicate that β-catenin mutations are uncommon in human lung, breast, kidney, cervical and ovarian carcinomas. To further explore other potential mechanisms of activating β-catenin signalling, we performed a mutational analysis in the $NH_2$-terminal regulatory region of the γ-catenin gene in these tumour cells. Only one potentially activating mutation was found in NCI-H1726 squamous cell lung carcinoma cells among 134 cell lines examined. This mutation resulted in Thr to Ile substitution at codon 19. Interestingly, the same amino acid substitution has been demonstrated at codon 41 in exon 3 of the β-catenin gene in 2 endometrial carcinoma tissues (Fukuchi et al, 1998). γ-catenin shares strong amino acid similarity with β-catenin, binds APC and Tcf, and exhibits signalling activity similar to that of β-catenin (Karnovsky and Klymkowsky, 1995; Rubinfeld et al, 1995; Sacco et al, 1995). γ-catenin mutation in H1726 cells might be involved in activation of the APC/β-catenin/Tcf signalling pathway. Three APC-associated proteins, β-catenin, γ-catenin and GSK-3β, have been linked to the APC/β-catenin/Tcf pathway by biochemical and genetic studies (Peifer, 1996; Kolligs et al, 2000). Sparks et al (1998) reported that no mutations of the γ-catenin and GSK-3β genes were detected in colorectal tumours lacking APC mutations. However, there have been very few reports on genetic alteration of these genes in a variety of human tumour cells. Our present results suggest that β-and γ-catenins may not be a frequent target of mutations involved in the pathogenesis of human lung, breast, kidney, cervical and ovarian carcinomas. If the role of the APC/β-catenin/Tcf-regulated transcription pathway is crucial for oncogenesis of these tumours, alterations in other genes that function in the Wnt signalling pathway must be elucidated.

### ACKNOWLEDGEMENTS

We are grateful to Dr C Korch, University of Colorado Health Sciences Center, Sequence Core, for his interest, advice and sequence analysis. We also thank J Jacobsen, C Dessev and E Li for their technical assistance. This study was supported by the University of Colorado SPORE in lung cancer (CA58187).

### REFERENCES

Barth AI, Nahke IS and Nelson WJ (1997) Cadherins, catenins and APC protein: interplay between cytoskeletal complexes and signaling pathways. *Curr Opin Cell Biol* **9**: 683–690

Behrens J, von Kries J, Kuhl M, Bruhn L, Wedlich D, Grosschedl R and Birchmeier W (1996) Functional interaction of β-catenin with the transcription factor LEF-1. *Nature (Lond)* **382**: 638–642

Calvo R, West J, Franklin W, Erickson P, Bemis L, Li E, Helfrich B, Bunn P, Roche J, Brambilla E, Rosell R, Gemmill RM and Drabkin HA (2000) Altered Hox and Wnt7a expression in human lung cancer. *Proc Natl Acad Sci USA* **97**: 12776–12781

Candidus S, Bischoff P, Becker KF and Hofler H (1996) No evidence for mutations in the α-and β-catenin genes in human gastric and breast carcinomas. *Cancer Res* **56**: 49–52

Fukuchi T, Sakamoto M, Tsuda H, Maruyama K, Nozawa S and Hirohashi S (1998) β-catenin mutation in carcinoma of the uterine endometrium. *Cancer Res* **58**: 3526–3528

Ilyas M, Tomlinson IPM, Rowan A, Pignatelli M and Bodmer WF (1997) β-catenin mutations in cell lines established from human colorectal cancers. *Proc Natl Acad Sci USA* **94**: 10330–10334

Karnovsky A and Klymkowsky MW (1995) Anterior axis duplication in *Xenopus* induced by the over-expression of the cadherin-binding protein plakoglobin. *Proc Natl Acad Sci USA* **92**: 4522–4526

Kawanishi J, Kato J, Sasaki K, Fujii S, Watanabe N and Niitsu Y (1995) Loss of E-cadherin-dependent cell-cell adhesion due to mutation of the β-catenin gene in a human cancer cell line, HSC-39. *Mol Cell Biol* **15**: 1175–1181

Kinzler KW and Vogelstein B (1996) Lessons from hereditary colon cancer. *Cell* **87**: 159–170

Kitaeva MN, Grogan L, Williams JP, Dimond E, Nakahara K, Hausner P, DeNobile JW, Soballe PW and Kirsch IR (1997) Mutations in β-catenin are uncommon in colorectal cancer occurring in occasional replication error-positive tumors. *Cancer Res* **57**: 4478–4481

Kolligs FT, Kolligs B, Hajra KM, Hu G, Tani M, Cho KR and Fearon ER (2000) Gamma-catenin is regulated by the APC tumor suppressor and its oncogenic activity is distinct from that of beta-catenin. *Genes Dev* **14**: 1319–1331

Morin PJ, Sparks AB, Korinek V, Barker N, Clevers H, Vogelstein B and Kinzler KW (1997) Activation of β-catenin-Tcf signaling in colon cancer by mutations in β-catenin or APC. *Science (Washington DC)* **275**: 1787–1790

© 2001 Cancer Research Campaign 

Oyama T, Kanai Y, Ochiai A, Akimoto S, Oda T, Yanagihara K, Nagafuchi A, Tsukita S, Shibamoto S, Ito F, Takeichi M, Matsuda H and Hirohashi S (1994) A truncated β-catenin disrupts the interaction between E-cadherin and α-catenin: a cause of loss of intercellular adhesiveness in human cancer cell lines. *Cancer Res* **54**: 6282–6287

Palacios J and Gamallo C (1998) Mutations in the β-catenin gene (CTNNB1) in endometrioid ovarian carcinomas. *Cancer Res* **58**: 1344–1347

Peifer M (1996) Regulating cell proliferation: as easy as APC. *Science (Washington DC)* **272**: 974–975

Peifer M (1997) β-catenin as oncogene: the smoking gun. *Science (Washington DC)* **275**: 1752–1753

Rubinfeld B, Souza B, Albert I, Munemitsu S and Polakis P (1995) The APC protein and E-cadherin form similar but independent complexes with α-catenin, β-catenin, and plakoglobin. *J Biol Chem* **270**: 5549–5555

Rubinfeld B, Robbins P, El-Gamil M, Albert I, Porfiri E and Polakis P (1997) Stabilization of β-catenin by genetic defects in melanoma cell lines. *Science (Washington DC)* **275**: 1790–1792

Sacco PA, McGranahan TM, Wheelock MJ and Johnson KR (1995) Identification of plakoglobin domains required for association with N-cadherin and α-catenin. *J Biol Chem* **270**: 20201–20206

Sparks AB, Morin PJ, Vogelstein B and Kinzler KW (1998) Mutational analysis of the APC/β-catenin/Tcf pathway in colorectal cancer. *Cancer Res* **58**: 1130–1134

*© 2001 Cancer Research Campaign*
